# First presentation with neuropsychiatric symptoms in autosomal dominant Alzheimer’s disease: the Dominantly Inherited Alzheimer’s Network Study

**DOI:** 10.1136/jnnp-2022-329843

**Published:** 2022-12-15

**Authors:** Antoinette O'Connor, Helen Rice, Josephine Barnes, Natalie S Ryan, Kathy Y Liu, Ricardo Francisco Allegri, Sarah Berman, John M Ringman, Carlos Cruchaga, Martin R Farlow, Jason Hassenstab, Jae-Hong Lee, Richard J Perrin, Chengjie Xiong, Brian Gordon, Allan I Levey, Alison Goate, Neil Graff-Radford, Johannes Levin, Mathias Jucker, Tammie Benzinger, Eric McDade, Hiroshi Mori, James M Noble, Peter R Schofield, Ralph N Martins, Stephen Salloway, Jasmeer Chhatwal, John C Morris, Randall Bateman, Rob Howard, Suzanne Reeves, Nick C Fox, for the Dominantly Inherited Alzheimer Network, Sarah Adams

**Affiliations:** 1 Dementia Research Centre, UCL Queen Square Institute of Neurology, London, UK; 2 UK Dementia Research Institute at UCL, London, UK; 3 Division of Psychiatry, University College London, London, UK; 4 Cognitive Neurology, Neurological Research Institute FLENI, Buenos Aires (Argentina), Buenos Aires, Argentina; 5 Department of Neurology, University of Pittsburgh Medical Center, Pittsburgh, Pennsylvania, USA; 6 Department of Neurology, Keck School of Medicine of the University of Southern California, Los Angeles, California, USA; 7 Department of Neurology, Washington University School of Medicine, St. Louis, Missouri, USA; 8 Department of Neurology, Indiana University School of Medicine, Indianapolis, Indiana, USA; 9 Department of Neurology, Asan Medical Center, University of Ulsan College of Medicine, Seoul, Korea (the Republic of); 10 Department of Pathology and Immunology, Washington University in St Louis MO, St Louis, Missouri, USA; 11 Division of Biostatistics, Washington University in St Louis MO, St Louis, Missouri, USA; 12 Department of Radiology, Washington University School of Medicine, Saint Louis, Missouri, USA; 13 Department of Neurology, Emory University School of Medicine Atlanta, Atlanta, Georgia, USA; 14 Department of Neuroscience, Icahn School of Medicine at Mount Sinai, New York, New York, USA; 15 Department of Neurology, Mayo Clinic Jacksonville, Jacksonville, Florida, USA; 16 German Center for Neurodegenerative Diseases (DZNE), Munich, Germany; 17 Munich Cluster for Systems Neurology, (SyNergy), Munich, Germany; 18 Department of Neurology, Ludwig-Maximilians Universität München, Munich, Germany; 19 German Center for Neurodegenerative Diseases, Tübingen, Germany; 20 Hertie Institute for Clinical Brain Research, University of Tübingen, Tübingen, Germany; 21 Osaka City University, Osaka, Japan; 22 Department of Neurology and Taub Institute for Research on Alzheimer's Disease and the Aging Brain, Columbia University, New York, New York, USA; 23 Neuroscience Research Australia, Sydney, New South Wales, Australia; 24 School of Medical Sciences, University of New South Wales, Sydney, New South Wales, Australia; 25 Sir James McCusker Alzheimer's Disease Research Unit, Edith Cowan University, Perth, Western Australia, Australia; 26 Department of Neurology, Butler Hospital & Alpert Medical School of Brown University, Providence, Rhode Island, USA; 27 Department of Neurology, Harvard Medical School, Boston, Massachusetts, USA

**Keywords:** ALZHEIMER'S DISEASE, BEHAVIOURAL DISORDER, COGNITION, NEUROPSYCHIATRY

## Introduction

Behavioural changes and neuropsychiatric symptoms (NPS) commonly occur in Alzheimer’s disease (AD) but may not be recognised as AD-related when they are the presenting feature. NPS are important as they are associated with greater functional impairment, poorer quality of life, accelerated cognitive decline and worsened caregiver burden.^1^

Autosomal dominant AD (ADAD), although <1% of total AD cases, provides a valuable opportunity to study the clinical heterogeneity of AD. The young age at onset reduces the prevalence of age-related comorbid pathologies and the near 100% penetrance of pathogenic mutations reduces the likelihood of misdiagnosis.^2^

Anxiety and depression commonly occur in ADAD family members, with increased levels of depression having been found among predementia female mutation carriers.^3^ Subsequent studies, however, have shown that anxiety and/or depression are common regardless of mutation status, occurring in almost one in three at-risk individuals, with one study reporting a higher rate of depression in non-carriers (17%) than asymptomatic carriers (5%).^4 5^ Despite the high frequency of NPS in ADAD families, relatively little is known about the proportion of ADAD cases who present with predominantly behavioural symptoms.

Our aims were to assess the first reported clinical change in symptomatic ADAD, to compare presentations across genotypes, and to compare cognitive performance between behavioural and cognitive-led presentations.

## Methods

Data from the first symptomatic visit of ADAD participants were obtained from Data Freeze 14 of the Dominantly Inherited Alzheimer Network (DIAN), an international multisite study of ADAD family members who are affected by, or at 50% risk of inheriting, pathogenic presenilin (*PSEN*) 1/2, or amyloid precursor protein (*APP*) mutations.^6^

For symptomatic participants, clinicians categorised the first predominant symptom as cognitive, behavioural, motor or unknown. For all symptomatic participants (regardless of presenting symptom domain), the first predominant behavioural symptom was identified. Cognitive function was assessed using a standardised neuropsychological test battery.^6^ ADAD mutation status was determined using Sanger sequencing.

Baseline demographics were compared using independent samples t-tests or Mann-Whitney U tests for continuous variables and χ^2^ or Fisher’s exact tests for categorical variables. Linear regression models with robust SEs that allowed for clustering within families compared cognitive performance (letter fluency, word list recall) between cognitive-led and behavioural-led presentations, adjusting for age, sex, disease duration and years of education. Binary logistic regression, where the outcome of interest was cognitive versus behavioural onset, was performed. Prespecified comparisons of interest were: (1) *PSEN1* versus *APP* and (2) *PSEN1* pre-codon200 versus *PSEN1* post-codon200 carriers; each analysis allowed for clustering within families. Proportions of first predominant behavioural symptom across genotypes and mutation subgroups were calculated.

Further details on study procedures and analyses are provided in online supplemental material.

10.1136/jnnp-2022-329843.supp1 Supplementary data

## Results

The dataset included 136 (23 *APP*, 113 *PSEN1/2*) carriers of whom 112 (82%) had predominantly cognitive onsets while 19 (14%) had behavioural-led presentations. Demographic details online supplemental table 1; demographics of genetic subgroups online supplemental tables 2 and 3).

There was no significant difference in age at onset between behavioural-led and cognitive-led presentations across all carriers (p=0.51) or across *PSEN1* carriers (p=0.80) but *PSEN1* precodon200 carriers were significantly younger than postcodon200 carriers (p=0.001).

Linear regression models, adjusted for age, gender, disease duration and years of education, found no significant difference in cognitive performance between cognitive-led and behavioural-led presentations: beta coefficient β_word immediate recall −0.06 (95% CI=−0.97 to 0.84, p=0.90), β_average verbal fluency −0.08 (95% CI −0.35 to 0.19, p=0.55).

Behavioural onset was more common among PSEN1 pre-codon200 carriers (n=8; 26%) than among pre-codon200 non-carriers (n=8;10%) (OR 3.14, 95% CI 1.08 to 9.11, p=0.036). There was no significant difference between *APP* and *PSEN1* carriers (OR 0.08, 95% CI 0.41 to 2.86, p=0.88).

**Figure 1:**
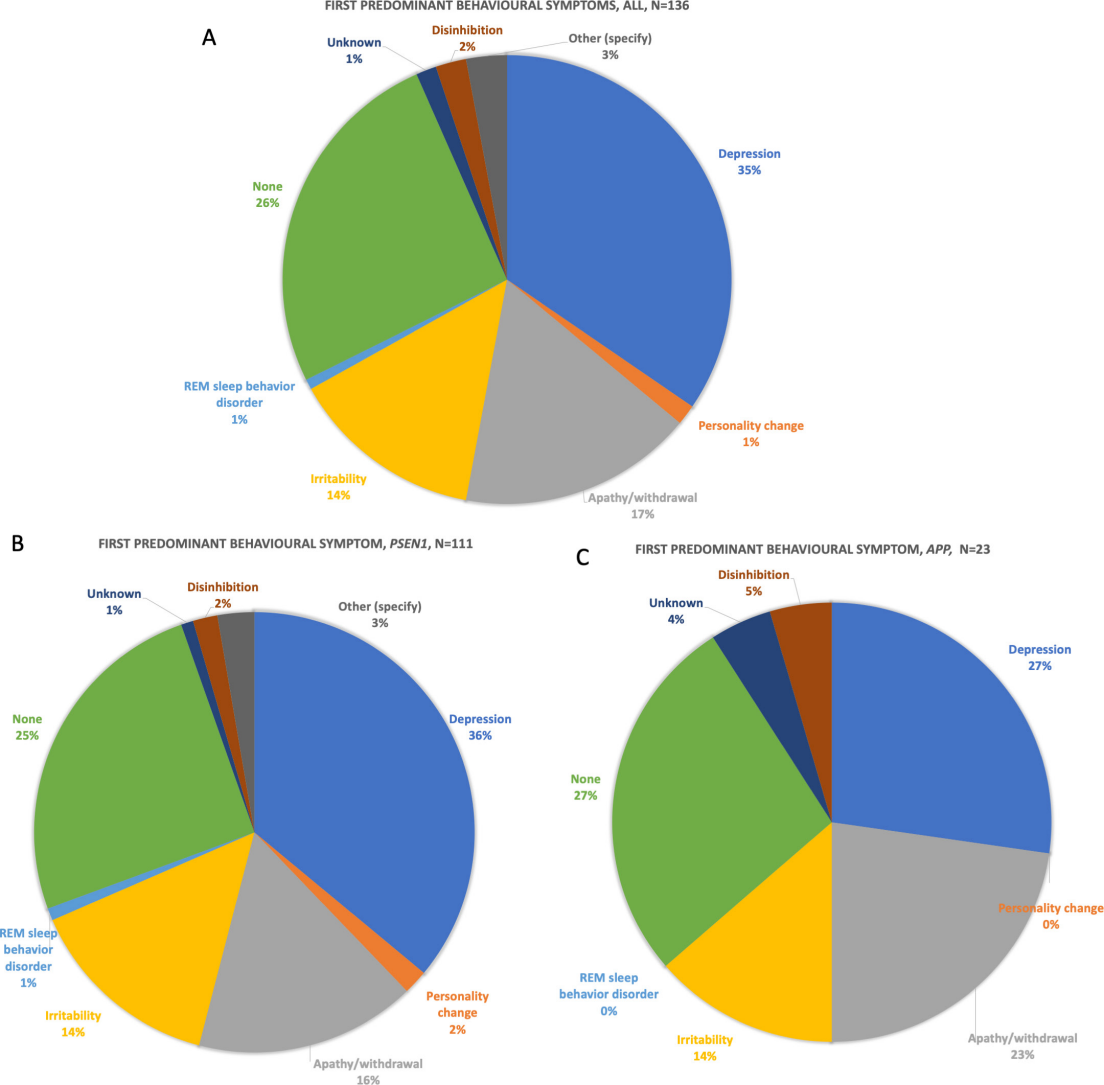
First predominant behavioural symptom reported. (A) displays data from all symptomatic mutation carriers, (B) symptomatic *PSEN1* carriers and (C) symptomatic *APP* carriers. There was no significant difference in the proportion of *PSEN1* and *APP* patients with depression as the first predominant behavioural symptom (p=0.80). APP, amyloid precursor protein; PSEN, presenilin.

The most commonly occurring first predominant behavioural symptom among all symptomatic carriers was depression, followed by apathy and irritability (figure 1).

## Discussion

Behavioural-led presentations, although less frequent than cognitive led, are relatively common in ADAD with 14% of cases presenting in this way. There were no significant differences in age at onset or cognitive performance between these two groups.

NPS occurred in over 60% of symptomatic carriers, with depression, apathy and irritability being especially common. Smaller DIAN series (n=58 and n=107 symptomatic carriers) previously found a reasonably similar frequency of behavioural/personality change.^4 7^ This is greater than the prevalence reported (approximately 40%) in the wider literature.^7^ This may be attributable to DIAN being a prospective study with active screening for these symptoms, which may have been under-reported in retrospective series.

The frequency (14%) of behavioural symptom onset is also higher than that reported in a large retrospective ADAD series (8%; n=17/213).^2^ Additionally, over 30% of cases reported here had a first predominant behavioural symptom of depression, followed by apathy (17%) and irritability (14%). Interpreting the clinical significance of these symptoms in ADAD is challenging: asymptomatic carriers were previously found to be less likely than non-carriers to experience behavioural changes.^4^ Nonetheless the high frequency of behavioural onset and NPS reported here suggests that these symptoms should be screened for as they may herald clinical onset.

There was no difference in the likelihood of behavioural predominant presentations between *APP* and *PSEN1* carriers, however pre-codon 200 *PSEN1* carriers were over three times more likely to have behavioural onset compared with post-codon 200 carriers. This is somewhat surprising given atypical cognitive presentations have been found to occur more commonly in post-codon 200 carriers.^2^ This result should be interpreted cautiously given the small numbers.

A limitation of this study is the reliance on clinician judgement of retrospective caregiver and participant reports to determine initial symptoms. However, recall bias is minimised by the prospective nature of this study as well as the performance of annual study visits for symptomatic participants. Individuals with NPS may be less likely to participate in multimodal observational research. However, this would, if anything, strengthen our findings regarding the high prevalence of non-cognitive symptoms/presentations. Finally, the relative rarity of ADAD resulted in small numbers being included in subgroup analysis.

## Conclusion

This paper shows the relatively high frequency of behavioural predominant presentations in ADAD, and describes the earliest NPS in this ‘genetically pure’ form of AD. Behavioural change and NPS are important, common and potentially under-recognised and undertreated features of ADAD, which may herald cognitive decline.

10.1136/jnnp-2022-329843.supp2 Supplementary data
