# Supplementary material for: First presentation with neuropsychiatric symptoms in autosomal dominant Alzheimer’s disease: the Dominantly Inherited Alzheimer’s Network Study
Source: J Neurol Neurosurg Psychiatry. 2022 Dec 15;94(5):403–5. doi: 10.1136/jnnp-2022-329843 (PMC10145026; doi:10.1136/jnnp-2022-329843)
Supplement: Supplementary data [file jnnp-2022-329843supp001.pdf]

Methods:

In cases where participants were symptomatic at the time of recruitment, data obtained at first visit were used. All participants identified a collateral information source, who was interviewed separately, to obtain a collateral history and for completion of the Clinical Dementia Rating® (CDR®) scale[1]. The scale includes information on day-to-day cognition from participant and informant. Participants were defined as symptomatic if (i) CDR was >0 and (ii) there was sustained cognitive decline (CDR score did not subsequently return to zero).

Subcategories of first predominant behavioural symptom included: apathy/withdrawal; depression; psychosis; disinhibition; irritability; agitation; personality change; “other behavioural or psychological symptoms”; rapid eye movement (REM) sleep behaviour disorder; no symptom; and “unknown”.

The Neuropsychiatric Inventory-Questionnaire (NPI-Q), an informant-based scale used to rate the presence and severity of symptoms in 12 behavioural domains, was also completed[2]. If neuropsychiatric symptoms were present in any given domain, the informant rated the severity as mild, moderate, or severe (scored 1–3, respectively) and scores were summed to obtain a total score (maximum 36).

A pre-specified comparison of the likelihood of behavioural as opposed to cognitive presentation in (i) *PSEN1* vs *APP*; and (ii) *PSEN1* pre-codon200 vs *PSEN1* post-codon200 carriers was conducted; these comparisons were of particular interest due to higher frequency of atypical presentations in (i) *PSEN1* compared to *APP* carriers and (ii) in post-codon200 compared to pre-codon200 carriers[3].

Analyses were carried out in Stata (version 16).

Table 1: Demographic details of behavioural and cognitive predominant presentations

	Behavioural predominant* N=19	Cognitive Predominant* N=112	P-value
Gender, n (%)[a]			0.77
Male	9 (47%)	49 (44%)	
Female	10 (53%)	63 (56%)	
Mutation type, n (%)[a]			1.00
APP	3 (16%)	19 (17%)	
PSEN1	16 (84%)	91 (81%)	
Pre-codon 200	N =8	N =22	
Post-codon 200	N= 8	N =69	
PSEN2	0 (0%)	<3 (2%)	
Age at onset, years (mean, (SD))[b]	41.4 (8.0)	42.8 (8.8)	0.51
Disease duration, years (mean, (SD)) [b]	2.9 (2.5)	2.8 (2.9)	0.83
Years of education[b]	14.2 (4.7)	13.5 (3)	0.41
CDR Global[b] (mean, (SD))	0.7 (0.2)	0.8 (0.6)	0.77
NPI-Q (mean, (SD))	6.1 (6.2)	4.7 (4.1)	0.49
Average Letter fluency (mean, (SD))	10.0 (3.4) N=18	10.2 (4.7) N=104	0.85
Word list recall (immediate) (mean, (SD))	3 (1.7) N=18	3 (2) N=104	0.94

[a]No significant difference in proportions on chi-square or fisher exact testing

[b]No significant difference between variables using Mann-Whitney U or independent sample t tests

*Two participants (1.5%) presented with motor symptoms, while the first predominant symptom was unknown in three cases.

Table 2: Demographic details of *APP* and *PSEN1* carriers.

	APP N=23	*PSEN1* N=111	P-value
Gender, n (%)[a]			
Male	11 (48%)	48 (43%)	0.69
Female	12 (52%)	63 (57%)	
Age at onset, years (mean, (SD))[b]	43.2 (7.9)	42.3 (8.7)	0.66
Disease duration, years (mean, (SD)) [b]	2.8 (2.7)	2.8 (2.9)	0.93

Demographic details for *PSEN2* carriers are not reported due to risk of unblinding.

[a]No significant difference in proportions on chi-square

[b]No significant difference between variables using independent sample t tests

Table 3: Demographic details of *PSEN1* precodon200 and postcodon200 carriers.

	PSEN1 precodon200 N=32	*PSEN1 postcodon200* N=79	P-value
Gender, n (%)[a]			
Male	15 (47%)	33 (42%)	0.62
Female	17 (53%)	46 (58%)	
Age at onset, years (mean, (SD))[b]	38.2 (7.2)	44.0 (8.7)	0.001
Disease duration, years (mean, (SD)) [c]	2.6 (2.4)	2.8 (3.0)	0.67

[a]No significant difference in proportions on chi-square

[b]Significant difference between variables using independent sample t test (p=0.001)

[c] No significant difference between variables using independent sample t test

References

1. Morris, J. C. The Clinical Dementia Rating (CDR): Current version and scoring rules. *Neurology* **43**, 2412–2412 (1993).

2. Kaufer, D. I. *et al.* Validation of the NPI-Q, a brief clinical form of the Neuropsychiatric Inventory. *J. Neuropsychiatry Clin. Neurosci.* **12**, 233–239 (2000).
3. Ryan, N. S. *et al.* Clinical phenotype and genetic associations in autosomal dominant familial Alzheimer's disease: a case series. *Lancet Neurol.* **15**, 1326–1335 (2016).
